# Supplementary material for: Transcriptional impacts of substance use disorder and HIV on human ventral midbrain neurons and microglia
Source: bioRxiv. 2025 Feb 8:2025.02.05.636667. Preprint. [Version 2] doi: 10.1101/2025.02.05.636667 (PMC11838593; doi:10.1101/2025.02.05.636667)
Supplement: 1 [file NIHPP2025.02.05.636667v2-supplement-1.pdf]

bioRxiv preprint doi: https://doi.org/10.1101/2025.02.05.636667; this version posted February 8, 2025. The copyright holder for this preprint (which was not certified by peer review) is the author/funder, who has granted bioRxiv a license to display the preprint in perpetuity. It is made available under aCC-BY 4.0 International license.

Supplementary Materials for

Transcriptional impacts of substance use disorder and HIV on human ventral midbrain neurons and microglia

Alyssa Wilson* *et al.*

*Corresponding author. Email: alyssa.wilson@mssm.edu

This PDF file includes:

Figs. S1 to S5
Tables S3, S6, S8

Other Supplementary Materials for this manuscript include the following:

Tables S1, S2, S4, S5, S7
Data S1 to S9

bioRxiv preprint doi: https://doi.org/10.1101/2025.02.05.636667; this version posted February 8, 2025. The copyright holder for this preprint (which was not certified by peer review) is the author/funder, who has granted bioRxiv a license to display the preprint in perpetuity. It is made available under aCC-BY 4.0 International license.

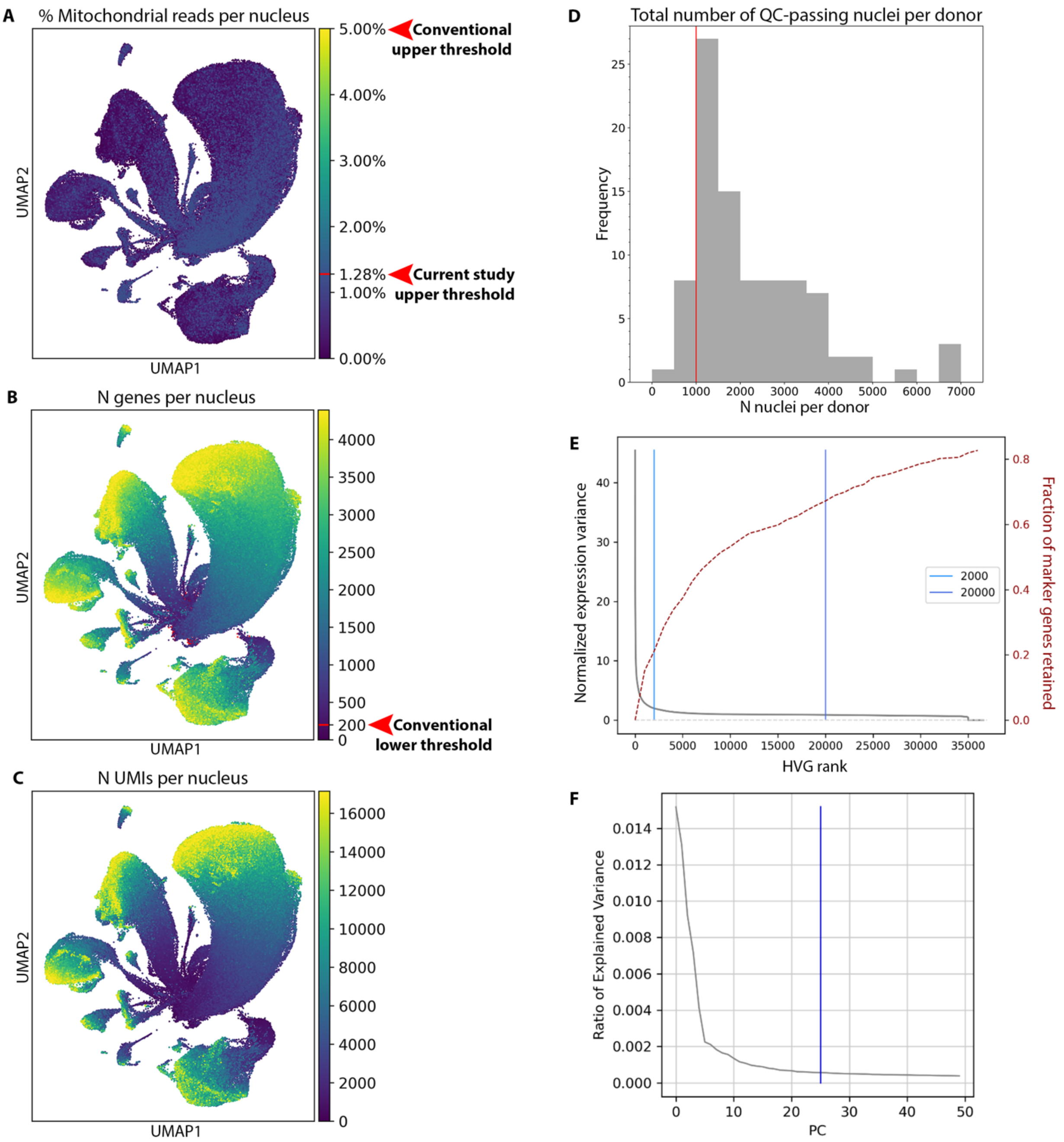

Fig. S1. Additional information about snRNA-seq QC and pre-processing.
(A)-(C) UMAPs showing values of QC heuristics, only for nuclei passing all QC nuclei. **(A)** Percent mitochondrial reads/nucleus. Red line/arrowhead at 1.28% shows the maximum value in our QC-passing nuclei; line at 5% shows a conventional filtering maximum. **(B)** Number of genes/nucleus; red line/arrowhead shows a conventional lower threshold of 200; QC-passing nuclei with <200 genes are colored red (see Fig. S2). **(C)** Number of UMIs/nucleus. **(D)** Distribution of the number of QC-passing nuclei/donor. Red line: the 1,000-nuclei threshold exceeded by 90% of donors. **(E)** Parameters used to determine the number of HVGs retained in analysis. Genes are ranked left to right by decreasing variability; black line (left y-axis) shows each gene's normalized expression variance; red line (right y-axis) shows the fraction of all genes ≤ that rank in our marker gene database. Blue lines show (right) our cutoff (20,000 HVGs) and (left) a conventional one (2,000 HVGs). **(F)** Explained variances of principle component analysis components ("PCs"); blue line shows the maximum component we retained, the 25th PC.

bioRxiv preprint doi: https://doi.org/10.1101/2025.02.05.636667; this version posted February 8, 2025. The copyright holder for this preprint (which was not certified by peer review) is the author/funder, who has granted bioRxiv a license to display the preprint in perpetuity. It is made available under aCC-BY 4.0 International license.

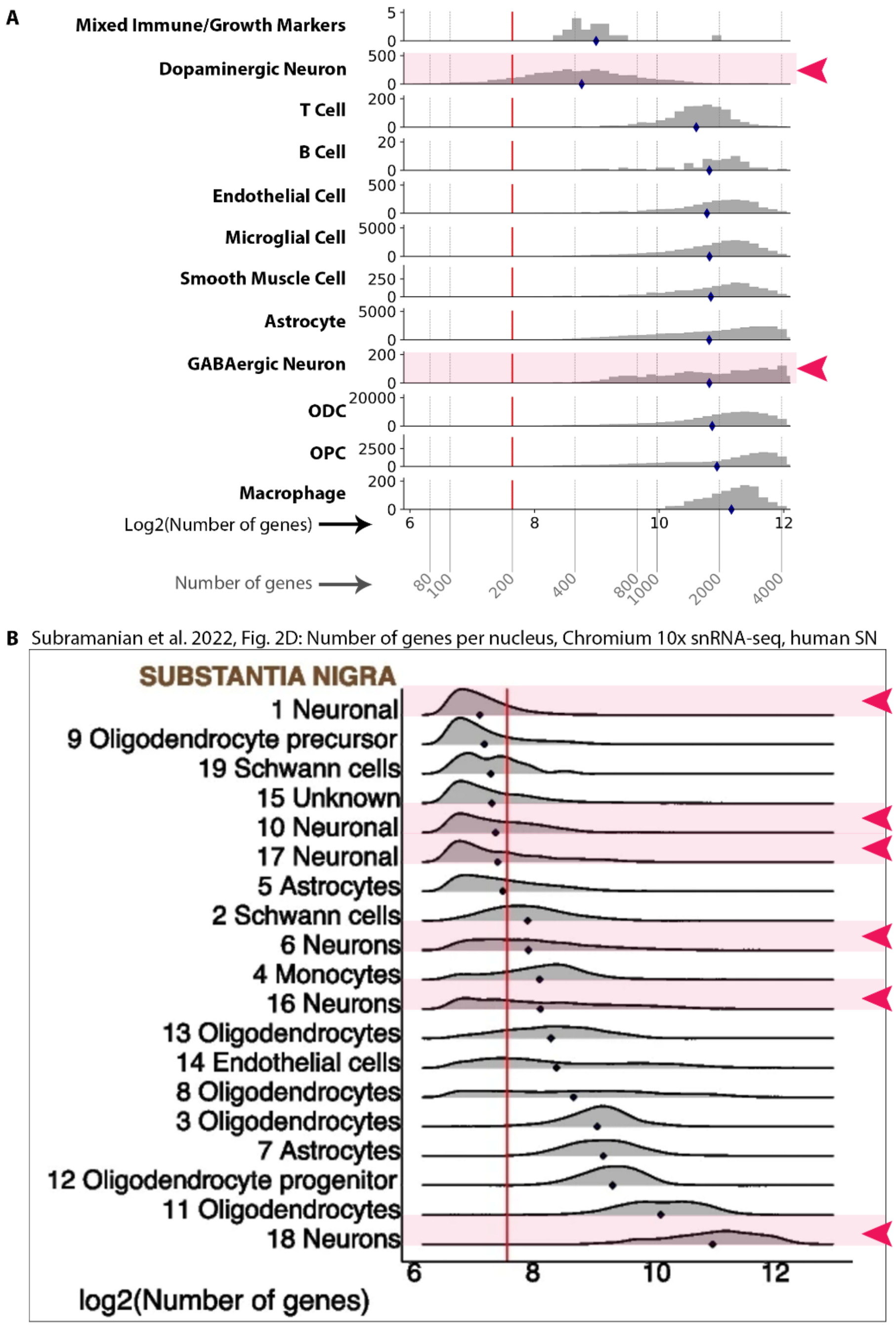

Fig. S2. Comparison of N genes/nucleus, by cell type, to published SN snRNA-seq data. **(A)** Histograms showing the log-transformed number of genes/nucleus, by cell type, for our QC-passing nuclei (**Figs. 1B**, **C**). Gray dashed lines show corresponding raw (non-log-transformed) gene counts. **(B)** Histograms showing log-transformed number of genes/nucleus for each cluster identified in previously published human SN snRNA-seq data, prepared similarly to ours (via Chromium 10x[1]). For **(A)** and **(B)**, neuronal cell types are marked with pink highlights and arrowheads, distribution means are indicated by blue diamonds, and red lines show the conventional lower threshold of 200 genes/nucleus.

bioRxiv preprint doi: https://doi.org/10.1101/2025.02.05.636667; this version posted February 8, 2025. The copyright holder for this preprint (which was not certified by peer review) is the author/funder, who has granted bioRxiv a license to display the preprint in perpetuity. It is made available under aCC-BY 4.0 International license.

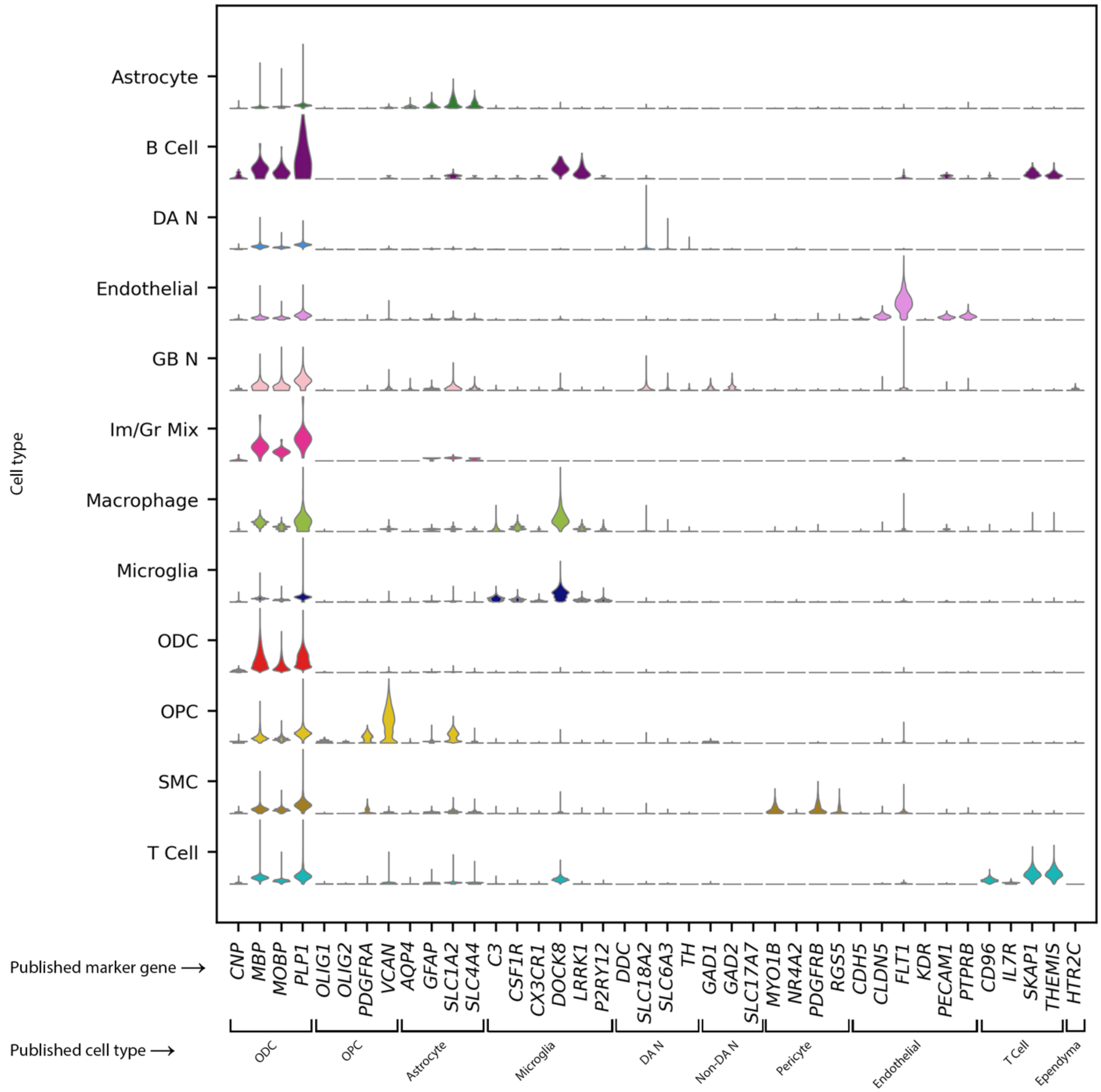

Fig. S3. Expression of previously published SN cell type marker genes, by cell type.
Expression in each of our cell types (rows) of previously identified (Wei et al. 2023[2]) marker genes for ventral midbrain cell types (columns).

bioRxiv preprint doi: https://doi.org/10.1101/2025.02.05.636667; this version posted February 8, 2025. The copyright holder for this preprint (which was not certified by peer review) is the author/funder, who has granted bioRxiv a license to display the preprint in perpetuity. It is made available under aCC-BY 4.0 International license.

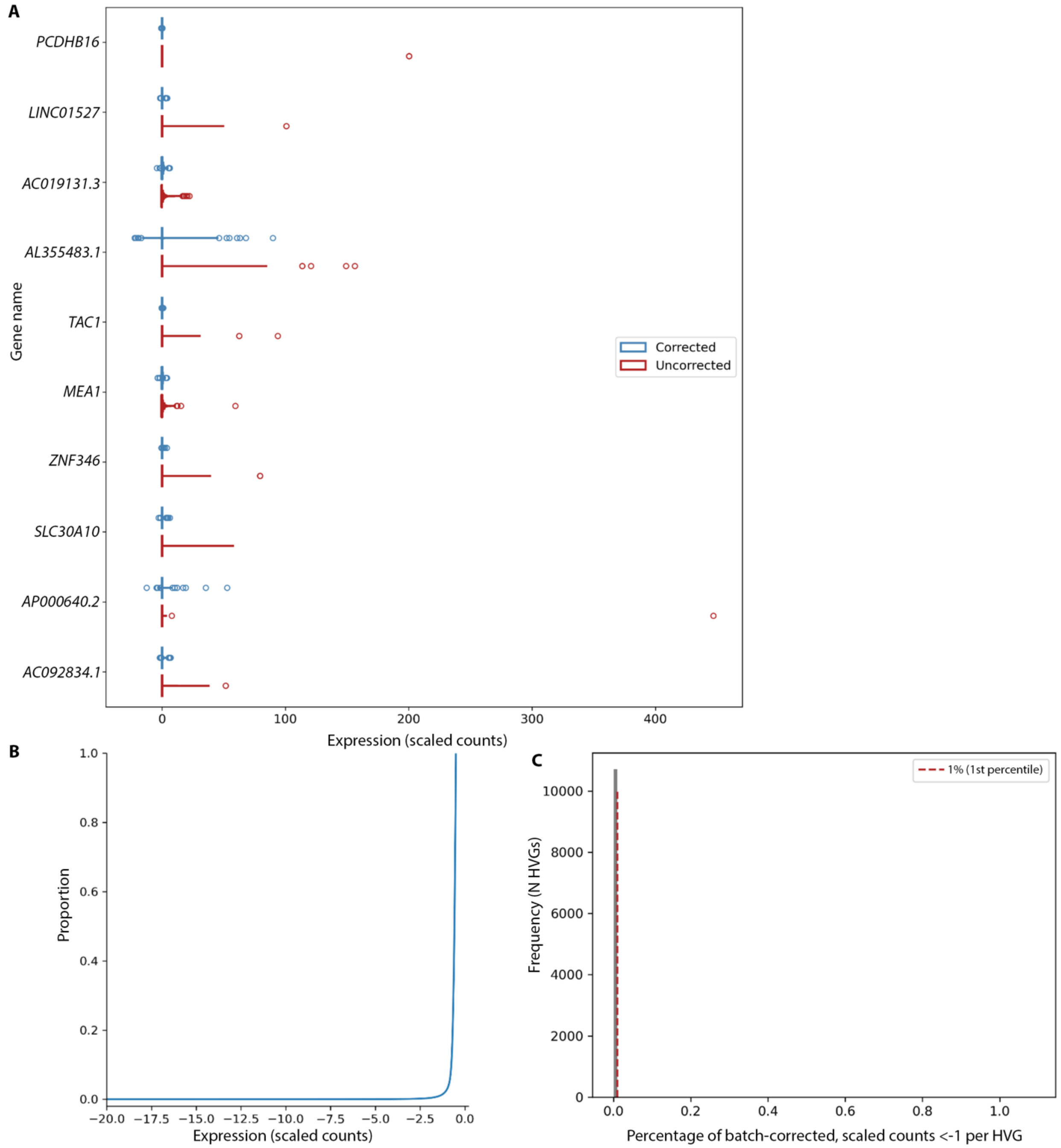

1521

1522 **Fig. S4. Impacts of batch correction on gene expression distributions for QCed data.**
1523 **(A)** Gene expression distributions before (red) vs. after (blue) batch correction, for 10 example
1524 HVGs selected randomly from our 20,000. **(B)** An empirical cumulative distribution showing the
1525 values of all batch-corrected, still-scaled read counts with values ≤ -0.5 (that would round to non-
1526 negligible negative counts ≤-1), highlighting a vanishing number of counts shifted to values <-2
1527 by batch correction. **(C)** Distribution showing the percentages of each HVG's batch-corrected
1528 counts with values <-1. Red line denotes 1% (corresponding to the lowest 1st percentile of an
1529 HVG's distribution).
1530

bioRxiv preprint doi: https://doi.org/10.1101/2025.02.05.636667; this version posted February 8, 2025. The copyright holder for this preprint (which was not certified by peer review) is the author/funder, who has granted bioRxiv a license to display the preprint in perpetuity. It is made available under aCC-BY 4.0 International license.

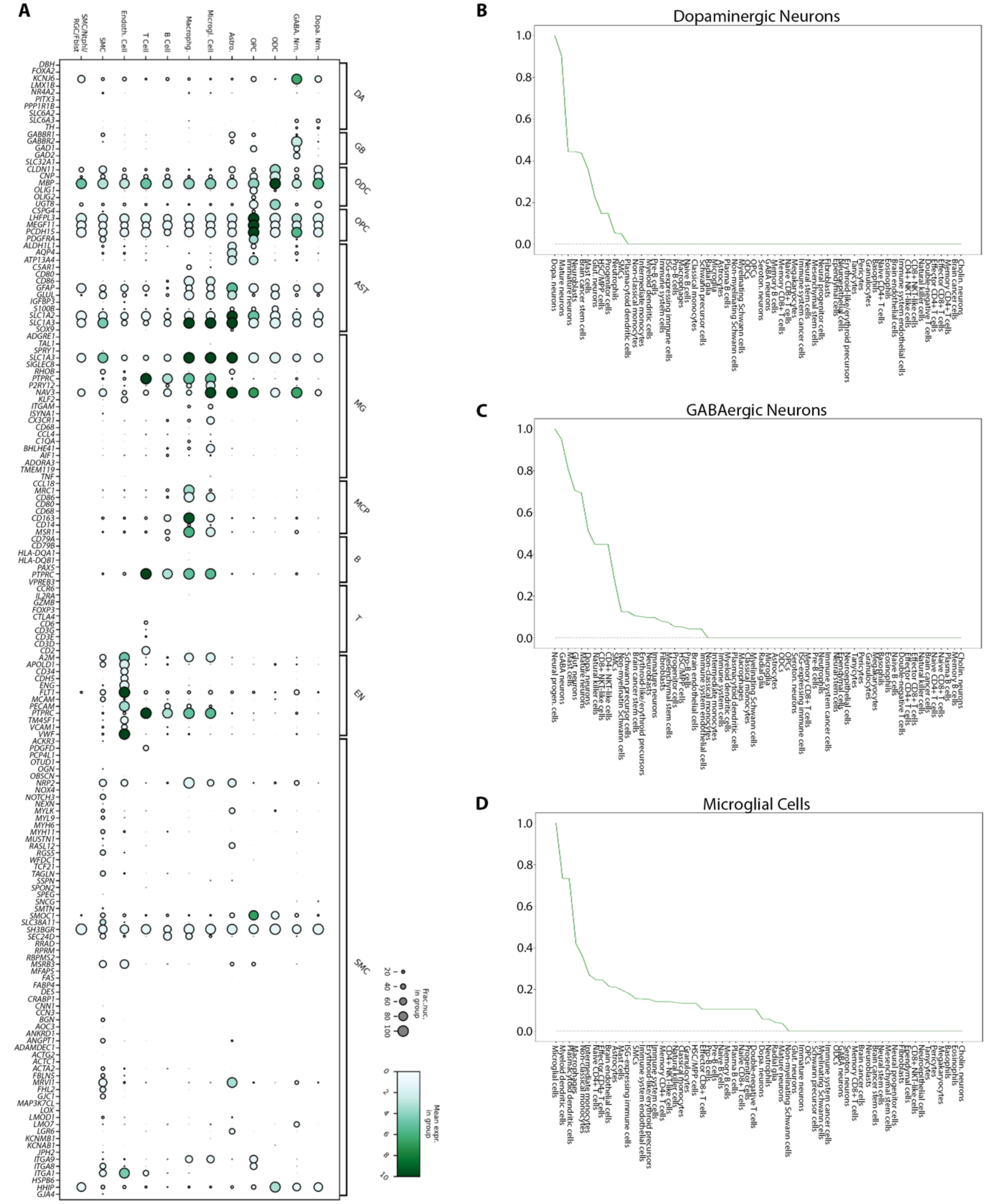

Fig. S5. Further information about likelihood-based cell typing of clusters.

(A) Dot plot showing, for each cell type in our dataset (top labels), expression of various expected SN cell type marker genes (left labels: expected marker genes, right labels: expected SN cell types). **(B)-(D)** Possible database (Data S6) cell types, ordered left to right by decreasing log-likelihood (and normalized to a maximum value of 1), for our **(B)** DA neuron, **(C)** GABA neuron, and **(D)** microglia clusters.

bioRxiv preprint doi: https://doi.org/10.1101/2025.02.05.636667; this version posted February 8, 2025. The copyright holder for this preprint (which was not certified by peer review) is the author/funder, who has granted bioRxiv a license to display the preprint in perpetuity. It is made available under aCC-BY 4.0 International license.

Table S1. (separate file)
Donor characteristics.

Table S2. (separate file)
Statistical comparisons of donor characteristics across donor groups. Bold text with asterisks indicates p<0.05. Double dashes indicate repeated comparisons or not-relevant self-comparisons.

Table S3.
Numbers of donors and nuclei per SUD drug class, by HIV status.

Table S4. (separate file)
snRNA-seq nuclear yields by donor and cell type.

Table S5. (separate file)
Statistical comparisons of cell type proportions across donor groups. Multiple-comparison-adjusted p-values are shown for Kolmogorov-Smirnov tests; bold and asterisks indicate p<0.05. The cell type proportions per donor group used in these comparisons are shown below this table.

Table S6.
DA neuron (DA)/microglia (MG) DEG subcluster compositions, for SUD+/HIV+u DEAs.

Table S7. (separate file)
Comparison of gene set enrichment results produced using Enrichr vs. GSEApy.

Table S8.
Fractions of significantly correlated DEG pairs in subclustering analyses.

bioRxiv preprint doi: https://doi.org/10.1101/2025.02.05.636667; this version posted February 8, 2025. The copyright holder for this preprint (which was not certified by peer review) is the author/funder, who has granted bioRxiv a license to display the preprint in perpetuity. It is made available under aCC-BY 4.0 International license.

Table S3. Numbers of donors and nuclei per SUD drug class, by HIV status.

**HIV status**	**N donors<br>N nuclei<br><br>cocaine SUD**	**N donors<br>N nuclei<br><br>opioid SUD**	**N donors<br>N nuclei<br><br>cocaine + opioid SUDs**	**N donors<br>N nuclei<br><br>neither SUD**	**Total N for HIV status**
**HIV-**	1<br>2,083	4<br>5,421	10<br>28,610	13<br>27,349	28<br>63,463
**HIV+u**	6<br>16,593	4<br>10,607	10<br>23,281	10<br>21,683	30<br>72,164
**HIV+d**	11<br>20,867	6<br>19,229	8<br>15,333	7<br>9,676	32<br>65,105
**Total N for SUD drug class**	18<br>39,543	14<br>35,257	28<br>67,224	30<br>58,708	90<br>200,732

bioRxiv preprint doi: https://doi.org/10.1101/2025.02.05.636667; this version posted February 8, 2025. The copyright holder for this preprint (which was not certified by peer review) is the author/funder, who has granted bioRxiv a license to display the preprint in perpetuity. It is made available under aCC-BY 4.0 International license.

Table S6. DA neuron (DA)/microglia (MG) DEG subcluster compositions, for SUD+/HIV+u DEAs.

DEA (effect)	Total N DEG subclusters	Median (IQR) N DEGs per subcluster	Median (IQR) subcluster mean similarity	N mixed DA/MG DEG subclusters	Median (IQR) N DEGs per mixed DA/MG subcluster	Median (IQR) mixed DA/MG subcluster mean similarity
**SUD+HIV- vs. SUD-HIV-**<br><br>**(SUD impacts without HIV)**	28	13 (7, 23)	0.88 (0.83, 0.94)	2	24 (20, 27)	0.91 (0.91, 0.92)
**SUD-HIV+u vs. SUD-HIV-**<br><br>**(HIV+u impacts without SUD)**	34	8 (5, 20)	0.94 (0.89, 0.95)	6	10 (7, 14)	0.90 (0.87, 0.92)
**SUD+HIV+u vs. SUD-HIV+u**<br><br>**(SUD impacts with HIV+u)**	28	12 (7, 21)	0.89 (0.85, 0.92)	6	16 (8, 38)	0.87 (0.80, 0.91)
**SUD+HIV+u vs. SUD+HIV-**<br><br>**(HIV+u impacts with SUD)**	27	9 (5, 22)	0.89 (0.85, 0.92)	3	9 (7, 23)	0.90 (0.87, 0.91)
**All**	117	10 (6, 21)	0.91 (0.86, 0.95)	17	12 (6, 20)	0.90 (0.86, 0.92)

bioRxiv preprint doi: https://doi.org/10.1101/2025.02.05.636667; this version posted February 8, 2025. The copyright holder for this preprint (which was not certified by peer review) is the author/funder, who has granted bioRxiv a license to display the preprint in perpetuity. It is made available under aCC-BY 4.0 International license.

Table S8. Fractions of significantly correlated DEG pairs in subclustering analyses.

Cell type mix	N DEAs analyzed	Median (IQR) fraction significantly correlated DEG pairs per DEA, across DEAs
**DA neurons, microglia**	4	0.30 (0.28, 0.30)
**GABA neurons, microglia**	2	0.27 (0.27, 0.28)
**DA neurons, GABA neurons**	2	0.46 (0.44, 0.49)
**All**	8	0.30 (0.28, 0.34)

bioRxiv preprint doi: https://doi.org/10.1101/2025.02.05.636667; this version posted February 8, 2025. The copyright holder for this preprint (which was not certified by peer review) is the author/funder, who has granted bioRxiv a license to display the preprint in perpetuity. It is made available under aCC-BY 4.0 International license.

Data S1. (separate file)

DEG lists, organized by cell type (DA neuron, GABA neuron, or microglia) and DEA type (impacts of HIV or SUD).

Data S2. (separate file)

Lists of Enrichr gene sets enriched by DEGs per DEA, cell type, and direction of dysregulation (up/down). Significantly enriched gene sets (with adjusted p-value <0.05; column E) are highlighted in green.

Data S3. (separate file)

Basic annotations for DEG subclusters, organized by cell type.

Data S4. (separate file)

Focused annotations for DEG subclusters, with references.

Data S5. (separate file)

UMAPs comparing expression data before (left) vs. after (right) batch correction, showing nuclei from one donor across pooled libraries ("batches"). Each plot title lists the donor's ID, and each library is shown in a different color.

Data S6. (separate file)

Cell type marker gene database used for cell typing.

Data S7. (separate file)

Heatmaps of DEG subclusters showing significant DEG pairwise Pearson correlations and related underlying data.

Data S8. (separate file)

Heatmaps showing mean normalized counts for each subcluster's DEGs, per case group donor, with donors labeled by SUD drug class (opioid, cocaine, or opioid+cocaine) and organized by their cross-DEG expression similarities (shown with a dendrogram above each heatmap).

Data S9. (separate file)

Zip file containing lists of GSEApy prerank gene sets enriched by each DEA's DEGs, organized by cell type. Files with names ending in "__all" include all gene sets having any DEGs; files ending with "__sig" include only significantly enriched gene sets.

bioRxiv preprint doi: https://doi.org/10.1101/2025.02.05.636667; this version posted February 8, 2025. The copyright holder for this preprint (which was not certified by peer review) is the author/funder, who has granted bioRxiv a license to display the preprint in perpetuity. It is made available under aCC-BY 4.0 International license.

References

1. Subramanian, A., Alperovich, M., Yang, Y. & Li, B. Biology-inspired data-driven quality control for scientific discovery in single-cell transcriptomics. *Genome Biol.* **23**, 267 (2022).

2. Wei, J. *et al.* Single nucleus transcriptomics of ventral midbrain identifies glial activation associated with chronic opioid use disorder. *Nat. Commun.* **14**, 5610 (2023).
